# Supplementary material for: Identification of photoperiod-induced specific miRNAs in the adrenal glands of Sunite sheep (Ovis aries)
Source: Front Vet Sci. 2022 Jul 22;9:888207. doi: 10.3389/fvets.2022.888207 (PMC9354845; doi:10.3389/fvets.2022.888207)
Supplement: Supplementary file 1 [file Data_Sheet_1.ZIP › Supplementary file/miRDeep2 file.zip!/miRDeep2 file/Know_Structure/LP42A1/oar-mir-374a.pdf]

miRBase precursor : oar-mir-374a
Total read count : 25298
oar-miR-374a read count : 20771
remaining reads : 4527

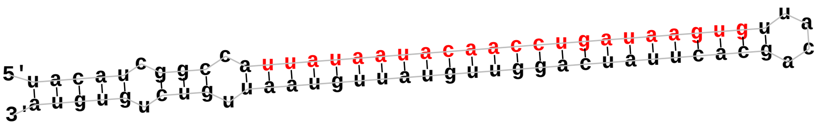

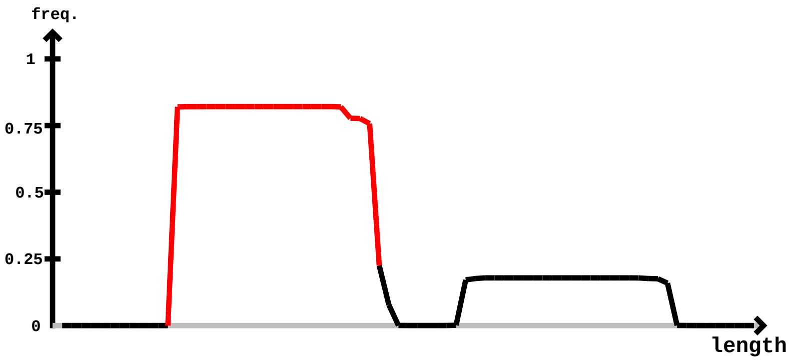


```
                oar-miR-374a
5'- uacaucggccauuauaauacaaccugauaaguguuacagcacuuaucagguuguauuguaauugucugugua   -3'    exp
    (((((.(((.((((((((((((((((((((((((....)))))))))))))))))))))))).))).)))))    reads      mm         sample
    ...........uuauaauacaaccugau............................................    24         0          seq
    ...........uuauaauacaaccugauU...........................................    17         1          seq
    ...........uuauUauacaaccugaua...........................................    9          1          seq
    ...........uuauaauacaUccugaua...........................................    2          1          seq
    ...........uuauaauacaaccugGua...........................................    4          1          seq
    ...........uuauaauacaacAugaua...........................................    2          1          seq
    ...........uCauaauacaaccugaua...........................................    3          1          seq
    ...........uuaAaauacaaccugaua...........................................    19         1          seq
    ...........uuaCaauacaaccugaua...........................................    15         1          seq
    ...........Cuauaauacaaccugaua...........................................    15         1          seq
    ...........uuauaauacaaccugaua...........................................    998        0          seq
    ...........uuaAaauacaaccugauaa..........................................    1          1          seq
    ...........uuauaauacaaccugauaa..........................................    23         0          seq
    ...........uuauaauacaaccugGuaag.........................................    2          1          seq
    ...........uuauaauacaaccugauaag.........................................    477        0          seq
    ...........uuaCaauacaaccugauaag.........................................    5          1          seq
    ...........uuauaauacaaccugauaagu........................................    13261      0          seq
    ...........uuaCaauacaaccugauaagu........................................    189        1          seq
    ...........uGauaauacaaccugauaagu........................................    2          1          seq
    ...........uuauaauacaaccugauaagug.......................................    3670       0          seq
    ...........uuaCaauacaaccugauaagug.......................................    52         1          seq
    ...........uGauaauacaaccugauaagug.......................................    2          1          seq
    ...........uuauaauacaaccugauaagugu......................................    1952       0          seq
    ...........uuauaauacaaccugauaaguguu.....................................    5          0          seq
    ...........uuauaauacaaccugauaaguguuacagc................................    1          0          seq
    ............uauaauacaaccugauaagu........................................    11         0          seq
    ............uauaauacaaccugauaagug.......................................    1          0          seq
    ............uauaauacaaccugauaagugu......................................    6          0          seq
    ............uauaauacaaccugauaaguguu.....................................    1          0          seq
    .............auaauacaaccugauaagu........................................    1          0          seq
    ..............uaauacaaccugauaagugu......................................    1          0          seq
    ..............uaauacaaccugauaaguguu.....................................    1          0          seq
    ...............aauacaaccugauaaguguuacagcacu.............................    2          0          seq
    ...............aauacaaccugauaaguguuacagcacuu............................    1          0          seq
    ........................................acuuaucagguuguauuAua............    2          1          seq
```

oar-miR-374a

```
uacaucggccauuauaauacaaccugauaagugguuacagcacuuaucagguuguauuguaauugucugugua
.......................................acuuaucagguuguauugua...........    1       0       seq
.......................................acuuaucagguuguauuguaau.........    11      0       seq
.......................................acuuaucagguuguauuguaauu........    11      0       seq
.......................................acuuaucagguuguauuguaauug.......    1       0       seq
........................................cuuaucagguuguauugu............    6       0       seq
........................................cuuaucagguuguauugua...........    49      0       seq
........................................cuuCucagguuguauugua...........    2       1       seq
........................................cuuaucagguuguauuguaa..........    20      0       seq
........................................cuuaucagguuguauuAuaa..........    1       1       seq
........................................cuuaucagguuguauuguaau.........    397     0       seq
........................................cuuaucagguuAuauuguaauu........    3       1       seq
........................................cuuaucagguuguauuguaauu........    3821    0       seq
........................................cuuaucagguuguauuguaauug.......    5       0       seq
........................................cuuaucagguuguauuguaauugu......    1       0       seq
.........................................uuaucagguuguauugua...........    1       0       seq
.........................................uuaucagguuguauuguaau.........    4       0       seq
.........................................uuaucagguuguauuguaauu........    118     0       seq
.........................................uuaucaAguuguauuguaauu........    2       1       seq
..........................................uaucagguuguauuguaau.........    1       0       seq
..........................................uaucagguuguauuguaauu........    60      0       seq
..........................................uaucagguuguauuguaauug.......    1       0       seq
..........................................uaucagguuguauuguaauugu......    5       0       seq
```
